# Current Approaches in Molecular Enzymology

**DOI:** 10.3390/life12030336

**Published:** 2022-02-24

**Authors:** Eszter Szabo, Attila Ambrus

**Affiliations:** Department of Biochemistry, Institute of Biochemistry and Molecular Biology, Semmelweis University, 1094 Budapest, Hungary; szabo.eszter1@med.semmelweis-univ.hu

Enzymes are the main executioners of living organisms. The quest for understanding their structure, function, and regulation is not only of intellectual interest but is also essential to elucidate the pathomechanisms of human diseases in order to find molecular targets and design possible pharmaceutical interventions. Today, an arsenal of biophysical and biochemical methods is available for enzymologists and the field is rapidly developing. The current Special Issue of *Life*, entitled “Current Approaches in Molecular Enzymology”, has been devoted to demonstrate the recent advances in protein science and the supporting methodology.

The research article by Gal et al. presents the first enzymological and genetic study of a Hungarian cohort (24 patients) with Pompe disease. A total of 15 different pathogenic or likely pathogenic acidic alpha-glycosidase variants were detected in homozygous or compound heterozygous form. Localization of the mutation sites correlated with residual enzyme activities and phenotypes. The authors emphasize the importance of the early diagnosis of the genetic disease in order to initiate the enzyme replacement therapy (accepted treatment since 2006) as soon as possible and avoid the accumulation of secondary pathological substances which would impair the response to the treatment [1]. From the same laboratory, a genetic study by Csaban et al. reports the occurrence of a heterozygous missense mutation in the *DLD* gene encoding the human lipoamide dehydrogenase (LADH) in patients diagnosed with Alzheimer’s disease (AD). LADH is the common E3 subunit of the 2-oxo acid dehydrogenase complexes; hence, it is crucial for mitochondrial function and in cellular metabolism. Since impaired mitochondrial function contributes to the pathogenesis of AD, the newly revealed LADH variant might represent a mild genetic risk factor for the disease [2]. T-cell leukemia virus type 1, 2, and 3 (HTLV 1, 2, and 3) are retroviruses causing several human diseases including adult T-cell leukemia. There is no standard therapy for HTLV infection yet, and potent molecules are in demand. Kassay et al. carried out a comparative analysis and inhibition profiling of HTLV proteases that are potential pharmacological targets [3]. Montioli et al. aimed to investigate RNase A dimers that are capable of entering cancer cells and inducing cell death via digesting intracellular RNA. The authors applied two protocols to induce self-association of RNase A and the resulting species (C- and N-swapped dimers) were investigated in terms of activity on RNA substrates, spectroscopic properties, and effects on cell viability when administered to MeWo and A375 human melanoma cell lines; the findings were correlated with the structures of the dimers. These results may gain importance in non-mutagenic antitumor therapies in the future [4].

Apart from these original research articles, three comprehensive reviews summarize our current understanding of the mechanistic and structural properties of proteins or protein families of great physiological and pathophysiological relevance. Nemeria et al. review the recent findings regarding the 2-oxoglutarate dehydrogenase and 2-oxoadipate dehydrogenase complexes that operate with specific E1 (E1o or E1a, respectively) and shared E2 and E3 subunits and were also shown to form hybrid multienzyme complexes; inter-subunit interactions were recently investigated in depth applying hydrogen/deuterium exchange mass spectrometry (HDX-MS) and MS coupled to chemical cross-linking [5]. Kovach provides a detailed description on the characteristics of serine proteases of the blood coagulation system and the importance of short proton bridges in catalytic efficiency. Kinetic deuterium isotope effects, proton inventory technique and low-field high-resolution nuclear magnetic resonance spectroscopy (^1^H NMR)—tools that proved to be particularly useful in the characterization of short proton bridges at binding sites near the active site and remote sites—are also outlined in the review [6]. Szöllősi guides us through the research of the last 2 decades that has aimed at understanding the structure and function of the transient receptor potential melastatin 2 (TRPM2) cation channel that also expresses ADP-ribose hydrolase activity (channel-enzyme, “chanzyme”) in invertebrates. The article also gives an introduction to the resolution revolution in single-particle cryo-electron microscopy (cryo-EM), which was essential to gain structural information about this complex transmembrane protein [7]. 

Three additional reviews intended to give insight into the background and recent advances of modern techniques that are now available for researchers not only to study the function and the structure of proteins, but also to manipulate and apply them for diagnostic or therapeutic purposes. The review by Ozohanics and Ambrus summarizes the basic concepts and recent developments of HDX-MS, a modern technique with a versatile applicability and no (or very high) size limit, making it capable of studying even protein complexes of MDa size [8]. The CRISPR-Cas system is widely known to be a useful tool for gene editing. Kwon and Shin provide a detailed overview of the applicability of the method in the diagnostics of selected infectious diseases. In comparison with the PCR-based detection systems, the CRISPR-Cas-based method provides a more time-efficient, less expensive solution that is easier to perform in the field with no need to compromise regarding sensitivity and specificity. All these qualities make this method a highly promising future solution also in the battle against the ongoing COVID-19 pandemic [9]. Finally, enzyme replacement therapy is a novel therapeutic approach for human diseases that is already approved for the treatment of lysosomal storage diseases. Specific proteins can be transferred into cells and cell organelles, even across the blood–brain barrier, upon fusing them to, e.g., a segment of the transactivator of transcription protein (TAT) of the human immunodeficiency virus (HIV). The review by Lichtenstein et al. demonstrates that the TAT-delivery system holds a huge therapeutic potential not only in restoring physiological protein/enzyme function in literally all kinds of mitochondrial disorders, but also in inducing apoptosis in tumor cells via selectively targeted (TAT-fused) proteins of the cellular apoptotic machinery [10].

We believe this Special Issue reached its goal to represent a collection of articles where the authors applied state-of-the-art technologies in the field of molecular enzymology. 

## Data Availability

Not applicable.
